# Comparison of 5 Versus 7-Day Ovsynch + Progesterone Releasing Intravaginal Device Protocols (PRID) and a Modified G7G with an Option of Heat Detection Protocol for 1st Service in Lactating Dairy Cows

**DOI:** 10.3390/ani11102955

**Published:** 2021-10-13

**Authors:** Christos Brozos, Evangelos Kiossis, Savvas Hatzieffraimidis, Anastasia Praxitelous, Ioannis Gouvias, Vasileios Kanoulas, Georgios Tsousis

**Affiliations:** 1 Clinic of Farm Animals, Faculty of Veterinary Medicine, Aristotle University of Thessaloniki, 54627 Thessaloniki, Greece; brozos@vet.auth.gr (C.B.); ekiossis@vet.auth.gr (E.K.); cdsavvas@vet.auth.gr (S.H.); praxitea@vet.auth.gr (A.P.); 2 CEVA Hellas, 16341 Ilioupoli, Greece; giannis.gouvias@ceva.com (I.G.); vasilis.kanoulas@ceva.com (V.K.)

**Keywords:** dairy cows, Ovsynch, progesterone, timed AI

## Abstract

**Simple Summary:**

The efficacy of two timed-AI protocols (5- and 7- Ovsynch + PRID) and a modified G7G protocol, that included intermediate heat detection, was evaluated. There was no difference in Pregnancy per AI between the two timed-AI protocols. The modified G7G protocol resulted in higher P/AI compared to the pooled data from the two TAI protocols. As a conclusion, enhancing detection of estrus within a synchronization protocol, by increasing the proportion of cows eligible to show estrus (e.g., by pre-synchronization) or by using activity-monitoring systems, could improve the reproduction indices of dairy cows.

**Abstract:**

The aim of this study was to evaluate the efficacy of two timed-AI (TAI) protocols (Group G5D, GnRH and PRID -5d- PGF2a -1d- PGF2a -1d- GnRH, *n* = 105 and Group G7D, GnRH and PRID-7d- PGF2a -1d- PGF2a -1d- GnRH, *n* = 98) and a modified G7G protocol combining heat detection (HD) and AI or TAI if HD failed (Group HD, GnRH and PRID -7d- PGF2a -1d- PGF2a -5d- HD or 5d TAI if no HD, *n* = 92). Pregnancy per AI (P/AI) did not differ between G5D and G7D protocol (G5D: 33.8% vs. G7D: 35.2%, P = 0.85). Cows assigned to G5D and G7D group were pooled as TAI group (GTAI) and further compared to GHD. Within the GHD, more primiparous cows exhibited estrous signs compared to multiparous cows (70.4% vs. 46.2%, P = 0.03). Furthermore, 49 cows (53.3%) were served after HD, whereas 43 cows (46.7%) were served after TAI. There was no difference in P/AI between cows served after HD (51.6%) or after TAI (43.0%, P = 0.49). GHD showed higher P/AI at 1st service compared to GTAI (49.1% vs. 36.4%, P = 0.04), whilst, median days to pregnancy did not differ between the two groups. Overall, P/AI of primiparous cows tended to be better in comparison with multiparous cows (48.3% vs. 37.2%, P = 0.06). In conclusion, there was no significant difference regarding the efficacy of 5- and 7-day Ovsynch + PRID protocols. Moreover, a modified G7G protocol, with intermediate heat detection, resulted in overall better P/AI compared to TAI protocols and appears as a promising strategy to optimize estrus detection for 1st AI.

## 1. Introduction

During the past decades, genetic progress, and the enhancement of management in dairy cattle have led to an increased milk production, Dry Matter Intake (DMI) and metabolism accompanied with an increased liver function. The enhanced metabolism can impair concentrations and half-life of reproductive hormones [1], leading eventually to reduced estrus expression and fertility. The development of synchronization protocols, that regulate ovulation and lead to Timed Artificial Insemination (TAI), has been used to achieve increased pregnancy rates in dairy cows [2].

The most popular protocols for TAI used for the first post-partum service are Presynch-Ovsynch [3], G6G or G7G and Double Ovsynch [4,5]. These or similar protocols are now the core of reproductive management programs in dairy cattle farms to optimize fertility and submission rate. Improvements of our knowledge on ovarian structures and the hormonal patterns that regulate them, has led to numerus modifications of the classical Ovsynch protocol proposed by Pursley, et al. [6], to further increase pregnancy rates. Such modifications include the addition of progesterone releasing intravaginal devices (PRID) at the time of the first GnRH administration, a PGF2α administration 5 or 7 days after the initial GnRH (together with PRID removal) and the addition of a second PGF2α 24 h apart [7,8].

The inclusion of a progesterone releasing intravaginal device in the Ovsynch protocol has positive effects on pregnancy per AI (P/AI). The increment of P4 concentration following PRID insertion has shown to increase P/AI especially in non-cycling or anestrus cows or cows with low progesterone at the time of PGF2α treatment of an Ovsynch protocol [9]. Hence, protocols have been developed that include PRID insertion between the first GnRH treatment and the PGF2α treatment and PRID removal concurrently with the PGF2α injection after 5 or 7 days. An addition of a second PGF2α treatment after 24 h, can increase the percentage of cows that present luteal regression and enhance synchronization efficiency [8]. Furthermore, this extra PGF2α treatment offers the possibility of hastening the time between the 2 GnRH injections, especially when the first one induces ovulation and leads to the formation of a new CL, as expected in pre-synchronization protocols [10]. Nevertheless, the efficiency of the 5- vs. the 7-day protocol for first post-partum AI has been studied only for a Co-Synch version [10]. Moreover, studies have shown that the ideal time frame of the estrus cycle for the initiation of the Ovsynch protocol is between 5 and 9 days [11]. A study conducted by Bello, et al. [12] has shown an increment in ovulatory response to the first GnRH, which improves the efficiency of the synchronization protocol, if the Ovsynch protocol starts during days 6 and 7 of the estrus cycle. Based on this knowledge, pre-synchronization protocols have been developed, such as the Double Ovsynch protocol, to further synchronize the stage of the estrus cycle at initiation of Ovsynch [5].

Nevertheless, AI after detection of estrus remains popular, especially in smaller farms. The wide implementation of precision dairy monitoring technology has increased the accuracy of estrus detection [13], making AI after natural estrus more efficient nowadays. However, Ovsynch protocols reduce estrus expression due to the 2nd GnRH injection which often leads to ovulation before estrus. As a result, Ovsynch protocols, applied to manipulate ovarian activity in high yielding dairy cows, and estrus detection, to optimize insemination success, could not be used concomitantly, but in a potential collaboration. This arrangement should be investigated to pose an appealing reproductive protocol.

The objective of this study was to investigate the efficacy of three hormonal protocols for the 1st post-partum (p.p.) AI in dairy cows. The basic purpose of the experiment was to compare a 5- vs. a 7-day Ovsynch + PRID protocol for their efficiency on the 1st p.p. TAI and the second to evaluate the efficacy of a modified G7G protocol with an option of an AI based on estrus detection.

## 2. Materials and Methods

### 2.1. Animals and Experimental Design

The experiment was performed in two commercial dairy farms (one in Macedonia and one in Thessaly region, Greece) including 200 and 140 milking cows, respectively, and an annual herd milk production of 10,500 and 11,500 kg of milk. Cows were housed in free stalls with unrestricted access to water and feed and were milked twice daily. Cows with reproductive disorders such as severe dystocia, clinical mastitis, or lameness were excluded from the study. 

**Figure 1:**
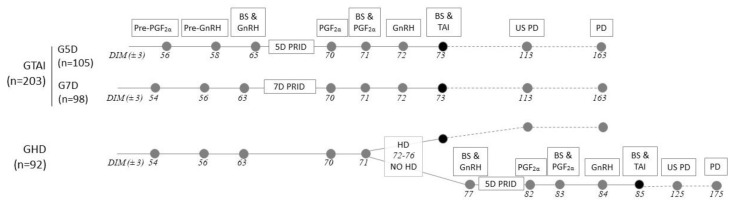
Timeline of the experimental design including two Timed Artificial Insemination (TAI) protocols (G5D and G7D) and a protocol combining AI after heat detection (GHD) with TAI if HD failed. BS = blood sample; US = transrectal ultrasonography; PD = pregnancy diagnosis; PRID = progesterone releasing intravaginal device.

At 50 days in milk (DIM), a total of 315 healthy cows were blocked by parity and randomly assigned to one of the three protocols (*n* = 105 per group), initiated on a weekly basis. Cows in group 5D and 7D, were assigned to a TAI protocol (Figure 1). Specifically, all cows received an intramuscular injection of 25 mg Dinoprost (5 mL of Cevaprost^®^, CEVA Santé Animal, Libourne, France) at 56 ± 3 DIM (G5D) and 54 ± 3 DIM (G7D), followed by one administration of 100 mg Gonadorelin (2 mL of Ovarelin^®^, CEVA Santé Animal, France) 2 days later (58 ± 3 for G5D, 56 ± 3 for G7D, pre-synch PGF2α and GnRH, respectively, Figure 1). Seven days later, a GnRH (2 mL of Ovarelin^®^) treatment and a progesterone releasing intravaginal device (PRID^®^, CEVA Santé Animal, France) was inserted in the animals of both groups. The PRID^®^ remained in situ for 5 (G5D) or 7 days (G7D). On the day of PRID^®^ removal (70 ± 3 DIM) and the following one (71 ± 3 DIM) all animals received 2 PGF2α injections (5 mL of Cevaprost^®^). Thirty-six hours after the second PGF2α administration (72 ± 3 DIM) cows were given a 2nd dose of GnRH (2 mL of Ovarelin^®^) and sixteen hours later (73 ± 3 DIM), timed artificial insemination (TAI) took place. 

Cows in Group HD (GHD, *n* = 105) from days 54 to 63 followed the pre- and synchronization protocol of G7D (Figure 1). These cows were not supplemented with the progesterone releasing intravaginal device. Seven and 8 days later (70 ± 3 and 71 ± 3 DIM, respectively) cows were injected with 5 mL of Cevaprost^®^. The following 5 days (72–76 ± 3 DIM), cows that were detected in estrus by the farm personnel were inseminated following the AM—PM rule. On day 77 ± 3 DIM, the cows of GHD that were not detected in estrus received a GnRH (2 mL of Ovarelin^®^) injection and a PRID^®^ was inserted. The device remained in place for 5 days, followed by two injections of PGF2α 24 h apart (82 and 83 ± 3 DIM). Thirty-six hours after the 2nd PGF administration (72 ± 3 DIM) cows received GnRH and TAI as previously described for the G5D and G7D. Pregnancy diagnosis was performed by transrectal ultrasonography with a 5-MHz linear-array transducer (Honda HS-101V; Honda electronics Co., Ltd., Toyohashi, Japan) at 33 ± 3 days post AI. A second pregnancy diagnosis was carried out via transrectal palpation at 80 ± 3 days post AI to detect pregnancy loss. All cows of G5D were eligible for analysis, whereas 7 cows were excluded from G7D (3 lost the devices, 2 became ill and 2 lacked sufficient documentation) and 13 cows were removed from GHD (5 due to healthy reasons and 8 due to insufficient documentation i.e., were lost in follow up from the farmers). Thus, the analysis was conducted with sample size of 105, 98 and 92 for G5D, G7D and GHD, resp.

### 2.2. Blood Collection and Hormonal Assay

A random subset of cows (*n* = 48, 50 and 38 for G5D, G7D and GHD, resp.) was used to collect blood samples from the coccygeal vein after disinfection of the area. The samples were stored on ice until centrifuged (3500× *g* for 15 min at 4 °C) within 60 min after collection, and then plasma was stored at −20 °C until hormone analyses. Blood samples were collected at: (a) day 63 or 65 ± 3 (Figure 1), and (b) day 71 ± 3, for cows from all groups, and (c) at TAI only for animals of G5D and G7D. Serum P4 concentration was evaluated by solid phase RIA, from unextracted sera, with the use of a commercially available radioimmunoassay kit (IMMUNOTEC, Prague, Czech Republic). The lower detection limit was 0.03 ng/mL, and the intra- and inter-assay coefficients of variation (CVs) was <10%. A cow with plasma P4 levels ≤1 ng/mL was documented as having no luteal activity (LA).

### 2.3. Statistical Analysis

Total sample size was estimated with G*power software (Heinrich-Heine-Universität Düsseldorf, Düsseldorf, Germany). The analysis was performed with Fischer’s exact test for two independent groups (GTAI and GHD). To conduct this analysis, we specified a difference of 15% in the P/AI (0.35 vs. 0.50), a significance level of 0.05, and a power of 0.80. Further statistical analysis was performed using the Statistical Analysis System v9.3 (SAS Institute, Cary, NC, USA). In a first step, differences in binary variables were evaluated by chi-square analysis (PROC FREQ). 

Generalized linear mixed models using PROC GLIMMIX were used to verify the effect of a set of variables on binary outcomes. The included fixed effects during modelling were group (G5D vs. G7D, for the initial analysis, and GTAI, which derived from pooling of G5D with G7D, vs. GHD, for the subsequent analysis), parity (primi- vs. multiparous), season (winter, November to April, vs. summer, May to October) and milk yield (low vs. high, based on median value of G5D and G7D, for the initial analysis, and of GTAI and GHD for subsequent analysis). Farm was included as a random effect. All two-way interactions with the group were included into the models. Backward stepwise selection with level of stay of 0.10 was used. Pairwise comparisons were performed using the LSMeans statement. Cows of group HD had different submission to AI compared to GTAI cows, as they were given two chances to be inseminated (after HD or TAI if HD failed). To adjust this effect, the rate of becoming pregnant and the median days to pregnancy for GHD and GTAI were evaluated using the Kaplan–Meyer survival curves (PROC LIFETEST) and Cox’s proportional hazards regression model (PROC PHREG). Due to our study design, that focused solely on 1st AI success, endpoint for a cow to become pregnant was set to 100 d. Results are presented as means or LSMeans ± SEM, unless stated otherwise. Differences were statistically significant at P < 0.05 and as a tendency at 0.05 ≤ P < 0.10.

## 3. Results

The mean and median lactation number and milk yield for all cows were 1.66 ± 0.03, 2 and 37.9 ± 0.43, 38 kg, respectively, and did not differ between the three groups (both P > 0.10). Overall P/AI was 38% (112 out of 295 cows) and pregnancy loss 8.9% (10 out of 112 cows, evenly distributed between groups).

### 3.1. Comparison of the Two TAI Protocols

Regarding the comparison between the two TAI protocols, there was no difference on P/AI (G5D: 33.8% vs. G7D: 35.2%, P = 0.85). Additionally, no significant interactions between protocols and parity, season or milk yield were detected (all P > 0.40). In these two groups of cows only parity tended (P = 0.06) to be different for P/AI (primiparous: 39.7% vs. multiparous cows: 26.9%, Table 1). G5D did not differ compared to group G7D regarding proportion of cows with no luteal activity at initiation of the protocol, after the 1st injection of PGF2α or at the time of AI (all P > 0.20, Table 2).

There was no difference in P/AI between cows with LA (*n* = 86) versus no LA (*n* = 12) at initiation of the protocol (38.4% vs. 33.3%, resp., P = 0.74). The same was true regarding cows with LA (*n* = 78) or no LA (*n* = 20) after the 1st PGF2α (41.0% vs. 25.0%, resp., P = 0.19). Cows with LA at AI (*n* = 16) tended to have lower P/AI compared to cows with no LA at AI (*n* = 82) (18.8% vs. 41.5%, resp., P = 0.08). Twelve cows (12.2%) showed no decrease in the P4 values from the day after the 1st PGF2α to AI (luteal regression). None of these cows conceived compared to 43% of the cows, in which a decrease in P4 values was observed (P = 0.004). The proportion of cows with no luteal regression was not influenced by treatment (15.5% in G5D vs. 15.2% in G7D, P = 0.96), by parity (18.4% in primi- vs. 12.3% in multiparous, P = 0.43) nor by milk yield (15.0% in low yielders vs. 15.7% in high yielders, P = 0.93). However, season tended to affect the proportion of cows with no luteal regression (8.2% in winter vs. 19.0% in summer, P = 0.10).

### 3.2. Comparison of TAI vs. HD-TAI Protocols

**Figure 2:**
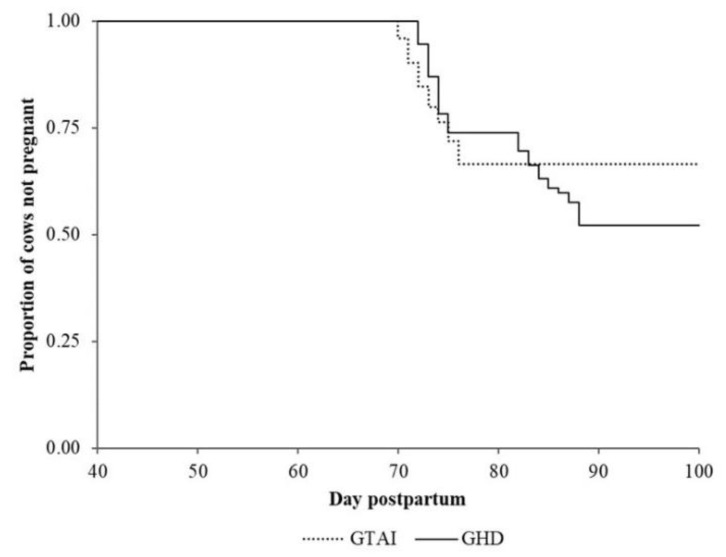
Kaplan–Meier survival curves for the proportion of cows not pregnant at 100 DIM in groups GTAI and GHD. Median days to pregnancy was 73 d for GTAI (95% confidence interval (CI) 71–75) and 75 d for GHD (95% CI 73–84). The rate of becoming pregnant was equal for GTAI compared to GHD (hazard ratio 1.4; 95% CI 0.9–2.0, P = 0.11).

Based on these findings, data from the two TAI protocols were pooled to form group TAI (GTAI, *n* = 203). GTAI showed lower P/AI compared to GHD (36.4% vs. 49.1%, resp., P = 0.04). Primiparous cows tended to have higher P/AI compared to multiparous cows (48.3% vs. 37.2%, resp., P = 0.06). Season and milk yield had no significant effect on P/AI (both P > 0.70). However, an interaction between season and treatment was evident (P = 0.09), as GTAI showed better P/AI in the summer (41.5%) compared to winter (31.2%) while cows in the GHD had better fertility in the winter compared to summer (54.3% vs. 44.0%, resp., Table 3). There was no difference between GTAI (*n* = 98) and GHD (*n* = 38) regarding the proportion of cows with no LA (12.2% vs. 13.2%, resp., P = 0.89) at initiation of the protocol. Regarding P4 values after the 1st PGF2α, GHD showed a higher proportion of cows with no LA (36.8%) compared to GTAI (20.4%, P = 0.05). The rate of becoming pregnant (hazard ratio 1.4; 95% CI 0.9–2.0, P = 0.11) and the median days to pregnancy (73 vs. 75, resp.) did not differ between GTAI and GHD (Figure 2).

Within Group HD, 49 cows (53.3%) exhibited estrus and were served, whereas 43 cows (46.7%) were not detected in estrus and received the PRID synch protocol for fixed-TAI. There was no difference in P/AI between the cows served after HD (51.6%) or after TAI (43.0%, P = 0.49). From the cows with no LA at initiation of the protocol (*n* = 5), 80% exhibited estrus (*n* = 4), whereas in cows with LA this proportion was 48.5% (17 out of 33, P = 0.19). After the 1st PGF2α, the proportion of cows with P4 < 1 ng/mL was equal in cows that were and were not detected in estrus (35.0% vs. 38.9%, P = 0.80). Within the HD group, more primiparous cows were detected in estrus compared to multiparous cows (70.4% vs. 46.2%, P = 0.03). No effect of season (61.2% in winter vs. 53.0% in summer, P = 0.45) or milk yield (56.5% in low yielders vs. 57.6% in high yielders, P = 0.92) was found regarding the proportion of cows detected in estrus.

## 4. Discussion

The results from the present study showed no differences between the efficiency of G5D and G7D TAI protocols for the first p.p. service. These findings agree with previous research, where similar P/AI was noted when comparing a 5D vs. a 7D Ovsynch protocols plus PRID for the resynchronization of dairy cattle [7]. Santos et al. [10], had previously detected an increment in P/AI in 5D compared to 7D TAI protocol. However, in this research, cows were submitted to AI at the same time of the second GnRH treatment (CoSynch) and only one injection of PGF2α was applied during the 7D CoSynch protocol. These differences in the study design could support the existing discrepancies. To our knowledge, there is no previous research comparing the efficiency of the 5D and 7D Ovsynch + PRID protocol in lactating dairy cows submitted to first service. Considering the above, it seems that reducing 2 days the interval from GnRH to the PGF2α treatment has no effect on follicle dominance and consequently P/AI, provided that an exogenous progestogen and an additional PGF2α treatment 24h later are administered. Decreasing the duration of the TAI protocol from 10 to 8 days could have a significant economic impact especially in large farms. However, the “5D plus progesterone” protocol appears to be more complicated in terms of compliance compared to the “7D plus progesterone” protocol, in which more treatments are applied at the same day of the week. Overall, only primiparous cows tended to show better P/AI compared to multiparous cows. Moreover, this effect was apparent both in the comparison of the 2 TAI protocols, as well as when group HD was added. This result was expected as synchronization rates and fertility are generally superior in first parity cows [14,15] probably due to lower milk production, dry matter intake and steroid metabolism.

The initiation of conventional Ovsynch during days 5 to 9 of the estrus cycle, when P4 concentration is expected to be elevated and a dominant follicle is likely present in the ovaries, increases the proportion of cows that ovulate in response to the first GnRH injection [11], which in turn promotes better synchronization rate and fertility [12]. Furthermore, low P4 concentrations during the initiation of an Ovsynch protocol impairs both synchronization [15] and fertility [16]. Cows included in our research, exhibited the same fertility regardless of P4 concentration at the initiation of G5D and G7D protocols. This could be attributed to the PRID supplementation, as fertility is improved with augmented concentrations of P4 present during the luteal phase, prior to PGF2α administration [17,18,19,20]. Moreover, the additional PGF2α treatment, 24h after the first one, seems necessary for complete luteolysis, as found by the high number (78 out of 98) of cows with luteal activity one day after the initial administration. The need of a second PGF2α treatment for successful luteolysis agrees with previous research that has validated this hypothesis [21]. However, even after the second PGF2α treatment, there was a considerable number of cows with high progesterone (>1ng/mL) at AI (16 out of 98) and with no decrease of P4 concentration (12 out of 98) between the two measurements (at the time of the first PGF2α treatment and at AI). Moreover, Vasconcelos, et al. [11], detected LA at AI in 15% of cows that initiated TAI protocol during early diestrus (days 5 to 9). Incomplete luteolysis was not influenced by parity nor milk yield, although, season seems to have a mild negative effect. The negative effect of elevated P4 on P/AI is well studied, and it has motivated researchers to develop innovative drug delivery systems, aiming an intermittent release of luteolytic doses of PGF, that mimic natural endometrial prostaglandin secretion [22]. 

The group HD exhibited better P/AI compared to TAI group. This is a logical consequence, since cows assigned to the HD group were first pre-synchronized to enhance estrus expression and then, had two opportunities to be inseminated (one after HD and one after failure of HD and application of a 5D TAI protocol). The insemination in almost 50% of the HD group was delayed, since many cows conceived after TAI. However, median days to pregnancy did not differ between GHD and GTAI (73 vs. 75 days). As a result, we believe that the combination of synchronization protocols with intermediate heat detection can lead to good fertility outcomes. Regarding P/AI, an interaction between season and treatment was evident. Based on the results (presented on Table 3), the lower P/AI in TAI group during winter was rather unexpected. Based on a further analysis we performed (data not shown), it was mainly attributed to the very disappointing P/AI (of only 7%) of low yielding multiparous cows of group TAI during winter. Typically, cows with low milk production have better reproductive efficiency [23,24]. However, in this case, we hypothesize that low milk yield could have the same underlying cause with low P/AI, which could be the severe winter conditions experienced during part of the experimental period [25]. The augmented levels of P4, observed in TAI group after PRID removal and first PGF2α administration, could be ascribed to a possible delayed clearance of progestogen originating from the PRID supplementation [26].

The results of the present study reveal that within the HD group, more primiparous cows exhibited estrus signs compared to multiparous cows, as it was expected [27]. Primiparous cows produce less milk [28] which promotes estrus expression [29]. Furthermore, low P4 at initiation of the synchronization protocol (day 63 of HD group) enhanced the detection of estrus. Probably, this was due to the presence of a young CL during that time, which responded well to the luteolytic effect of the second PGF2a treatment seven days later, as well as to the presence of young follicles able to produce sufficient amounts of estradiol and consequently, promote heat expression [30,31]. Based on this finding, if HD is a part of the reproductive management, the initiation of the G7G modified protocol should take place closer to the pre-synch protocol.

## 5. Conclusions

Only half of the cows in the HD group exhibited and were served after estrus. However, these cows were offered an immediate second chance of insemination and as a result they presented greater P/AI compared to solely TAI group. Additionally, this practice resulted in no difference regarding median days to pregnancy among all groups. Consequently, improving heat detection efficiency via investing in precise monitoring technologies or by increasing the proportion of cows eligible to show estrous (by a pre-synchronization protocol) would probably improve fertility in dairy farms. Furthermore, primiparous cows tend to express estrus more efficiently and are excellent candidates for a reproductive management based on heat detection. However, if a TAI protocol is preferred for ovulation synchronization, “5D plus progesterone” protocol seems to be equally efficient with “7D plus progestogen” protocol.

## Figures and Tables

**Table 1:** LS means of pregnancy per AI (P/AI) at 1st service and P-values of the variables under consideration for the comparison between groups 5D and 7D.

Variable	Class	*n*	P/AI, %	*F*-Value	*p*-Value
Treatment				0.04	0.85
	G5D	105	33.8		
	G7D	98	35.2		
Season				1.93	0.17
	Summer	77	39.3		
	Winter	126	29.7		
Parity				3.46	0.06
	LN = 1	72	39.7		
	LN > 1	131	26.9		
Milk Yield				0.02	0.89
	Low (<37.3 kg)	101	35.0		
	High (≥37.3 kg)	102	34.0		

**Table 2:** Proportion of cows with P4 ≤ 1 ng/mL at initiation of the protocol, after the 1st injection of PGF2α and at the time of AI in cows synchronized with a 5- and a 7-day protocol (Group 5D, *n* = 48 and 7D, *n* = 50, respectively).

Time Point and Variable	Group 5D	Group 7D	*p*-Value
Initiation of protocol			
P4 ≤ 1 ng/mL (%)	10.4	14.0	0.59
After 1st PGF2a			
P4 ≤ 1 ng/mL (%)	18.8	22.0	0.69
At AI			
P4 ≤ 1 ng/mL (%)	79.2	88.0	0.24

**Table 3:** LS means of pregnancy per AI (P/AI) at 1st service and P-values of the variables under consideration for the comparison between groups TAI and HD.

Variable	Class	*n*	P/AI, %	*F*-Value	*p*-Value
Treatment				4.27	0.04
	GTAI	203	36.4		
	GHD	92	49.1		
Season				0.00	0.95
	Summer	114	42.3		
	Winter	181	42.7		
Parity				3.50	0.06
	LN = 1	99	48.3		
	LN > 1	196	37.2		
Milk Yield				0.13	0.72
	Low (<38 kg)	141	43.7		
	High (≥38 kg)	154	41.4		
Treatment × Season				2.74	0.09
	GTAI Summer	77	41.5		
	GTAI Winter	126	31.2		
	GHD Summer	37	44.0		
	GHD Winter	55	54.3		

## Data Availability

Data sharing not applicable.
